# Does krill oil enhancing the new bone formation in orthopedically expanded median palatal suture in rat model? A micro-CT and immunohistochemical analysis

**DOI:** 10.1186/s12903-024-04644-5

**Published:** 2024-07-29

**Authors:** Doga Simsek, Gulay Dumanli Gok, Sibel Demirci Delipinar

**Affiliations:** 1 Department of Orthodontics, Faculty of Dentistry, Biruni University, Merkezefendi Mahallesi G/75 Sokak, No: 1-13, Zeytinburnu, Istanbul, Turkey; 2 Department of Orthodontics, Faculty of Dentistry, Istanbul Medipol University, Göztepe Mahallesi, Ataturk Caddesi, No: 40/16, Beykoz, Istanbul, Turkey; 3 Department of Histology and Embriology, Faculty of Medicine, Biruni University, Merkezefendi Mahallesi G/75 Sokak, No: 1-13, Zeytinburnu, Istanbul, Turkey

**Keywords:** Krill oil, New bone formation; Rapid maxillary expansion, Rat

## Abstract

**Background:**

The purpose of this study was to assess the effects of systemically given krill oil (KO) on the development of new bone formation in the sutura palatina media following rapid maxillary expansion (RME).

**Methods:**

28 4–5 week-old male Wistar albino rats were randomly divided into 4 groups: Control (C), Only Expansion (OE) (no supplement but undergoing expansion and retention), KE (supplemented during both the expansion and retention phases), Krill Oil Nursery Group (KN) (supplemented during the 40-day nursery phase as well as during the expansion and retention phases). A 5-day RME was followed by a 12-day retention period. All rats were euthanized simultaneously. Micro-computerized tomography (Micro-CT), hemotoxylen-eosin (H&E) staining, and immunohistochemical analysis were conducted. Kruskal-Wallis and Dunn tests with Bonferonni corrrection were applied (*p* < 0.05).

**Results:**

Expansion and KO supplementation did not cause a statistically significant change in bone mineral density (BMD), bone volume fraction (BV/TV), spesific bone surface (BS/BV) and trabecular thickness (Tb.Th). While the expansion prosedure increased the trabecular seperation (Tb.Sp), KO supplemantation mitigated this effect. The KE group exhibited a statistically significantly increase in trabecular number (Tb.N) compared to the OE group. Although receptor activator of nuclear factor-kappa-Β ligand (RANKL)/osteoprotegerin (OPG) ratios did not show significant differences between groups, the KE and OE groups demonstrated the lowest and highest value, respectively. KE showed a reduced amount of tartrate-resistant acid phosphatase (TRAP) compared to the OE.

**Conclusion:**

KO positively affected the architecture of the new bone formed in the mid-palatal suture. In this rat model of RME, results support the idea that administering of KO during the expansion period or beginning before the RME procedure may reduce relapse and enhance bone formation within the mid-palatal suture.

## Background

Oppenheim’s statement [1] in 1934, “Retention is the most difficult problem in orthodontia; in fact, it is the problem,” remains highly accurate even now in many cases, especially in those requiring palatal expansion [2, 3]. Since the maxilla is related with 10 bones in the facial skeletal system, the major impediment to the separation of the maxillary halves comes not only from the mid-palatal area but also from the other sutures that surround maxilla and mandibular condyles. It has been reported that reaction forces causing relaps of the expansion dissipate within the craniofacial skeleton over at least 6 weeks and in the condyle region for 3 weeks [4, 5]. To overcome this problem, a retention period of at least 6 months is advised [6].Additionally, numerous appliances have been designed and many treatment modalities have been tried with the aim of preventing relapse. Ultimately, scientists have highlighted the impact of the functional reorganization of bone formation within the intermaxillary suture during the initial phases of relapse following expansion [7, 8]. However, prolonged post-expansion retention periods cause poor oral hygiene, related deminerilazation of teeth, and peri-miniscrew enfections. Enhancing the production and growth rate of new bone within the mid-palatal suture could serve to impede arch width regression and reduce the duration of retention. For this purpose, researchers have concentrated on strategies to expedite the formation of new bone using various approaches, such as laser stimulation, herbal extracts, antioxidants, vitamins, nanotechnological materials and medical agents [2].

Driven by their observed ability to stimulate bone metabolism and their anti-inflammatory mechanism by lowering levels of pro-inflammatory cytokines, long-chain polyunsaturated n-3 fatty acids (PUFA) have the potential to be the subject of research in this context. However, only a few researchers have investigated the effects of omega-3s on tooth movement experimentally [9–11], and their effect on RME has not been studied.

PUFAs such as eicosapentaenoic acid (EPA) and docosahexaenoic acid (DHA) are found mainly in fish. Due to the limited availability of fish as a resource, there is an increasing desire to explore alternative sources of n-3 PUFAs. Krill are small sea crustaceans found in polar seas, obtained from the largest of the Antarctic shrimp-like zooplankton, *Euphausia Superba*. Surprisingly, unlike fish oil, 100% Krill oil (KO) is a unique substance, more abundant in terms of EPA and DHA ( 39.29–80.69%) and contains naturally occuring minor bioactive components such as astaxanthin, sterols, tocopherols, vitamin A, flavonoids, and minerals [12]. Additionally, KO’s lipid form is phospholipid, which has greater bioavailability than fish oil, which contains triglycerides [13]. Researcers mostly concentrate on KO because of their role in maintaining psychological and physiological health, their immunomodulatory and antiinflamatory effects, and their abilitity to stimulate bone metabolism by restraining osteoclastic activity and promoting osteoblastic activity [14]. Thus, we hypothesized that KO could have potential as a promoter of accelerating new bone formation in expanded mid-palatal suture. In the present study, a rat model was established with the aim of evaluating the effects of KO on the quality and quantity of the skeletal maxillary expansion and reducing relapse in the expanded-palatal suture, beyond what has been previously reported.

## Methods

This research was carried out according to National Research Council’s Guide for the Care and Use of Laboratory Animals on 28 4–5 week-old male Wistar albino rats, with a body weight ranging from 130 to 150 g. The rats were kept in plastic cages, fed a standard pellet diet, given unlimited access to water. To determine the appropriate sample size for studying new bone formation on midpalatal suture, G*Power Software version 3.1.9.2 was utilized. Based on the reference study [7], the sufficient sample size was calculated as 7 for each group, totaling 28, by determining the effect size as (f) = 0.894 for 80% power with 95% confidence. The 28 rats were randomly divided into 4 equal groups as follows:

Control (C): Saline was administrated during the observation period of 57 days.

Only expansion (OE) group: Saline was administrated during the 40-day nursery period and the 17-day expansion and retention period.

Krill Oil Expansion (KE) group: Saline was administrated during 40-day nursery period, and KO was administered only during the 17-day expansion and retention period.

Krill Oil Nursery Group (KN): KO was administered during nursery phase of 40 days and during the 17-day expansion and retention period.

The dose is equally related to body weight, although it is not the only factor that influences the scaling for dose calculation. The correction factor (K_m_) is estimated by dividing the average body weight (kg) of the species by its body surface area (m^2^) [15]. In present study, according to data obtained from FDA quidelines, the average human body weight and body surface area were assumed to be 60 kg and 1.62 m^2^, respecively. Similary, the surface area for a 150 gr rat was assumed to be 0.025 m^2^ [16]. Therefore, the K_m_ factors for human and rats were calculated as 37 and 6, respectively. The K_m_ ratio (6,17 kg/m^2^) was obtained by dividing human K_m_ factor by the animal K_m_ factor. The animal equivalent dose (AED) was calculated on the basis of body surface area by either dividing or multiplying the human dose (mg/kg) by the K_m_ ratio as follow:

AED (mg / kg) = Human does (mg / kg) × K_m_ ratio.

According to the prospectus, the recommended daily human dose is 1192 mg/kg, equivalent to 2 capsules of Neptun^®^ Krill Oil (Neptune Technologies and Bioresources, Quebec, Canada). Considering AED equation, the rat dose was determined to be 122.64 mg/kg. The KO capsules were opened with a scalpel, and their contents were diluted in saline to a ratio of 5 mg/kg. The equivalent rat dose of KO was administrated by gavage. The weights of the rats were measured daily, and according to this method, an 200 g rat was administrated 24,5 mg of KO diluted in 1 mg of saline.

**Fig. 1:**
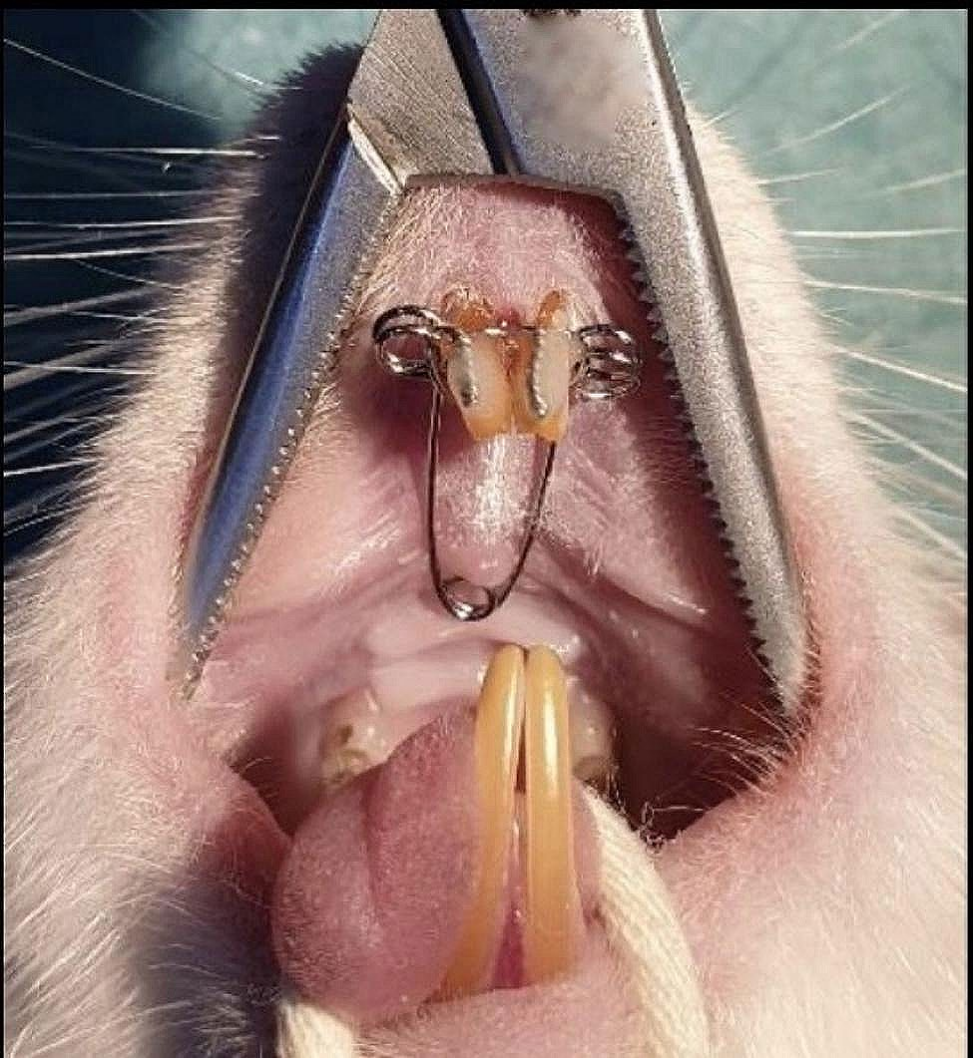
Experimental design

The V shaped expansion devices were bent from 0.012-inch steel orthodontic wire with a 13 mm arm length, a 10 mm distance between the 2 outer arms, and 2 mm diameter helix springs in the area corresponding to the V bend and the distal part of the arms. The activated appliance was adjusted to the anterior teeth of the rats by preparing retention grooves to apply 30 g of force (Fig. 1) These applications were performed under general anesthesia with 90/10 mg/kg xylazine (Xylazinbio %2, Bioveta, Ankara, Turkey) / ketamine (Ketalar, Vem Ilac, Cankaya, Ankara ). During the 5-day expansion period, the appliance was activated once at the beginning of the period. This single activation allows for controlled and consistent expansion of the mid-palatal suture, mimicking the clinical scenario of RME. Expansion of the maxillary suture was observed, and subsequently appliance was fixed for a 12-day retention period. At the end of the retention period, the rats were given 90/10 mg/kg xylazine / ketamine, and euthanasia was performed by taking blood from the heart. The heads of the sacrificed rats were separated from their bodies with a sharp scissors, and the maxillae were dissected and fixed. Immunohistochemical evaluation and micro-computed tomography (micro-CT) analysis were performed.

The scanning of the samples was performed in vitro using a micro-CT (Bruker micro-CT, SkyScan 1174, Kontich, Belgium) device operated at 50 kV and 100 µA. With the assistance of a 1.3-megapixel camera, cross-sectional slices of 1304 × 1024 pixels resolution were obtained from each sample with a pixel size of 17 μm. A 0.5 mm Al filter was employed during sample scanning, utilizing a vertical axis rotation of 360 degrees, a camera exposure time of 1400 ms, and a rotation step of 0.6 degrees. The expanded palatal suture and bilateral bones area were selected as region of interests with dimensions of 2.40 mm–0.90 mm-0.60 mm for quantification of the new bone features in terms of bone mineral density (BMD), bone volume fraction (BV/TV), spesific bone surface (BS/BV), trabecular thickness (Tb.Th), trabecular seperation (Tb.Sp), trabecular number (Tb.N) determinants.

The specimens were fixed using 4% buffered neutral formaldehyde (Atabay Kimya, Istanbul, Turkey) for 24 h. Following fixation, the tissues were decalcified in Morse’s decalcification solution consisting of 5% formic acid and 20% sodium citrate, which was renewed every other day for 30 days. The decalcified samples were dehydrated by passing through a rising series of 70%, 90%, 96% and 100% ethyl alcohol (Merck, Darmstadt, Germany) for 24 h each. Tissues were passed through toluene (Riedel-de Haën, Seelze, Germany) 3 times for 5 min for transparency and placed in an oven at 56 °C for 45 min each in soft paraffin (46–48 °C) and hard paraffin (56–58 °C) for infiltration. After routine tissue follow-up was completed, the specimens were embedded in hard paraffin, and the blocks were allowed to harden at room temperature. 5-µm-thick sections were obtained using a rotary microtome (HM 340E, Thermo-Fisher Scientific, Massachusetts, USA). These sections were deparaffinized and stained with Hematoxylin & Eosin (H&E). Microphotographs were obtained at x2 magnification using an Olympus BX-61 light microscope with DP-72 camera attachment. Palatal suture width was assessed using the Image-J 1.53q program at the point corresponding to the upper first molar’s mid-coronal plane.

For immunohistochemical examinations, 5-µm-thick sections were placed onto poly-l-lysine-coated slides (Sigma, St. Louis, MO, USA) and incubated overnight at 40 °C. After deparaffinization in toluene and rehydration in graded alcohol series, antigen retrieval was conducted by heating in a microwave oven for 3–5 min. The sections were washed with phosphate buffer, kept in 3% hydrogen peroxide, kept in the UV block for 5 min, and incubated 1 night at 4 °C with the primary antibodies. To show angiogenesis, vascular endothelial growth factor (VEGF) primary antibody (S-53,462, Santacruz Biotechnology, Heidelberg, Germany) was applied. To demonstrate bone remodeling, osteoprotegerin (OPG) (PA586053), receptor activator of nuclear factor kappa B (RANK) (PA588904) and receptor activator of nuclear factor kappa B (RANKL) (MA516156) (Thermo Fisher Scientific, Massachusetts, USA) primary antibodies were applied. The sections were washed, dried and the secondary antibody was applied and left for 30 min. Subsequently, they were kept in streptavidin-peroxidase and 3,3’-diaminobenzidine (DAB) for color development. The sections were counterstained with Mayer’s hematoxylin and mounted with glycerol gelatin (Sigma, St. Louis, MO, USA). All incubations were performed in a humidity chamber at room temperature, using PBS for washes between the incubation steps.

The photographs were transferred to a computer, and the staining intensity of antibodies in the sections was calculated for each group using FIJI Image-J 1.53q software to avoid visual bias. The program divided the digitized into 3 histogram profiles: DAB, hematoxylin, and an additional image after selecting the staining type by color deconvolution. The hematoxylin and additional image profiles that were not to be used were turned off, while the threshold level was determined on the remaining DAB profile. The threshold level ranged from 0 to 255. The most appropriate threshold level was selected for each section. When determining the threshold level, the selected areas were stained with red color. After an average threshold level value was determined, the ‘Apply’ tab was clicked on the program, and the selected areas were painted black. The program counted the cells stained in black and gave the percentage of each section stained with antibody.

For tartrate resistant acid phosphatase (TRAP), the samples were treated with xylene and alcohol and then washed with distilled water. The TRAP staining was performed following the procedure recommended by the manufacturer of the TRAP Kit (Acid Phosphatase, Leukocyte, Sigma-Aldrich, Germany). Semi-quantitative evaluation was performed with the histochemical scoring (H-SCORE) method in five randomly selected regions of each tissue sample at x40 magnification with a Nicon Eclipse E200 light microscope and a Nikon DS-Fi2 camera. Image-J 1.53q program were used for analysis.

### Statistical analysis

Kruskal-Wallis test was applied for comparisons between groups. In case of a significant difference between the groups, Dunn’s test with Bonferroni correction was utilized. *p* < 0.05 was considered statistically significant (IBM SPSS Statistics 25).

## Results

### Micro-CT results

**Fig. 2:**
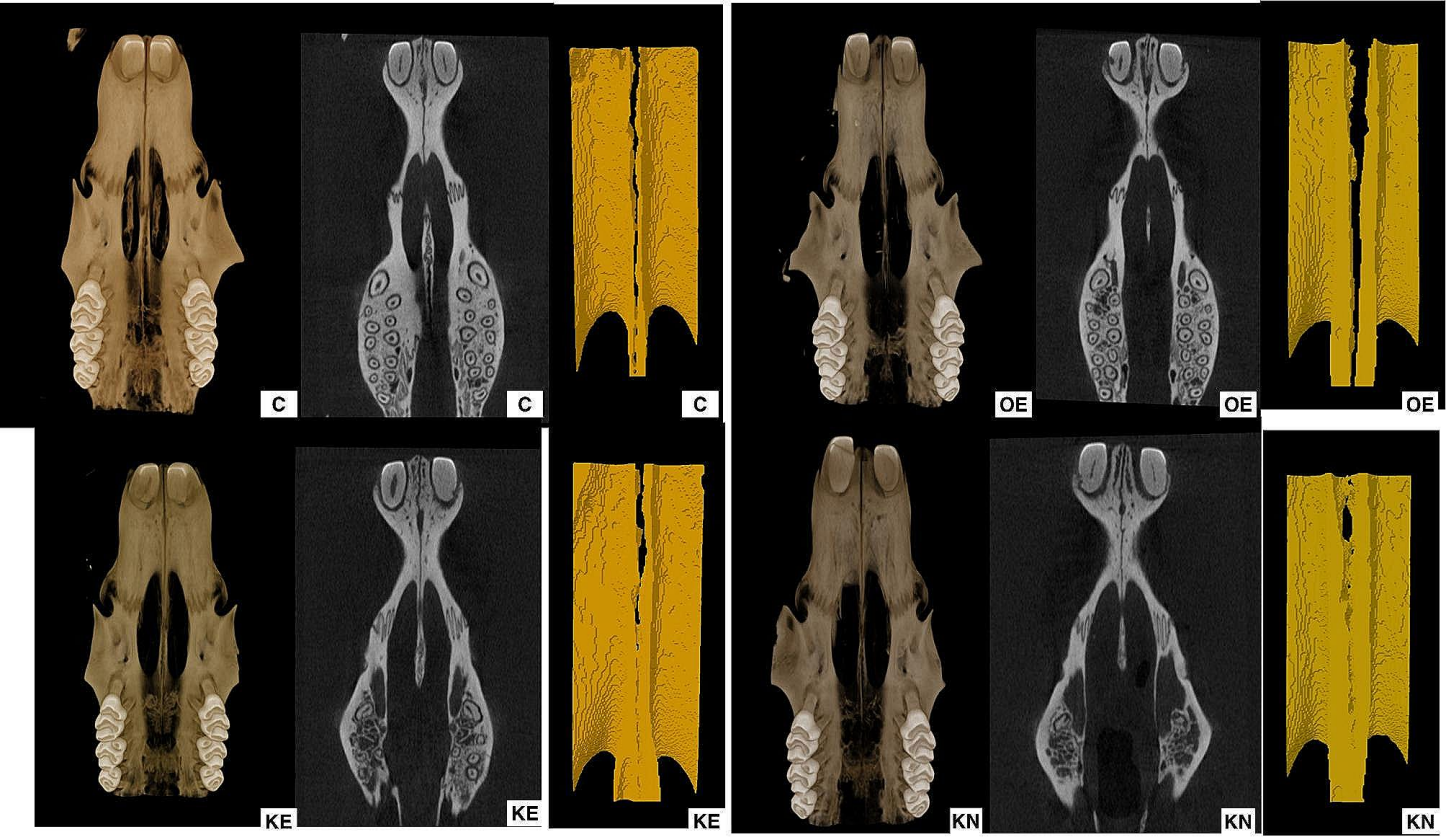
Micro-CT view of opened mid-palatal suture

**Fig. 3:**
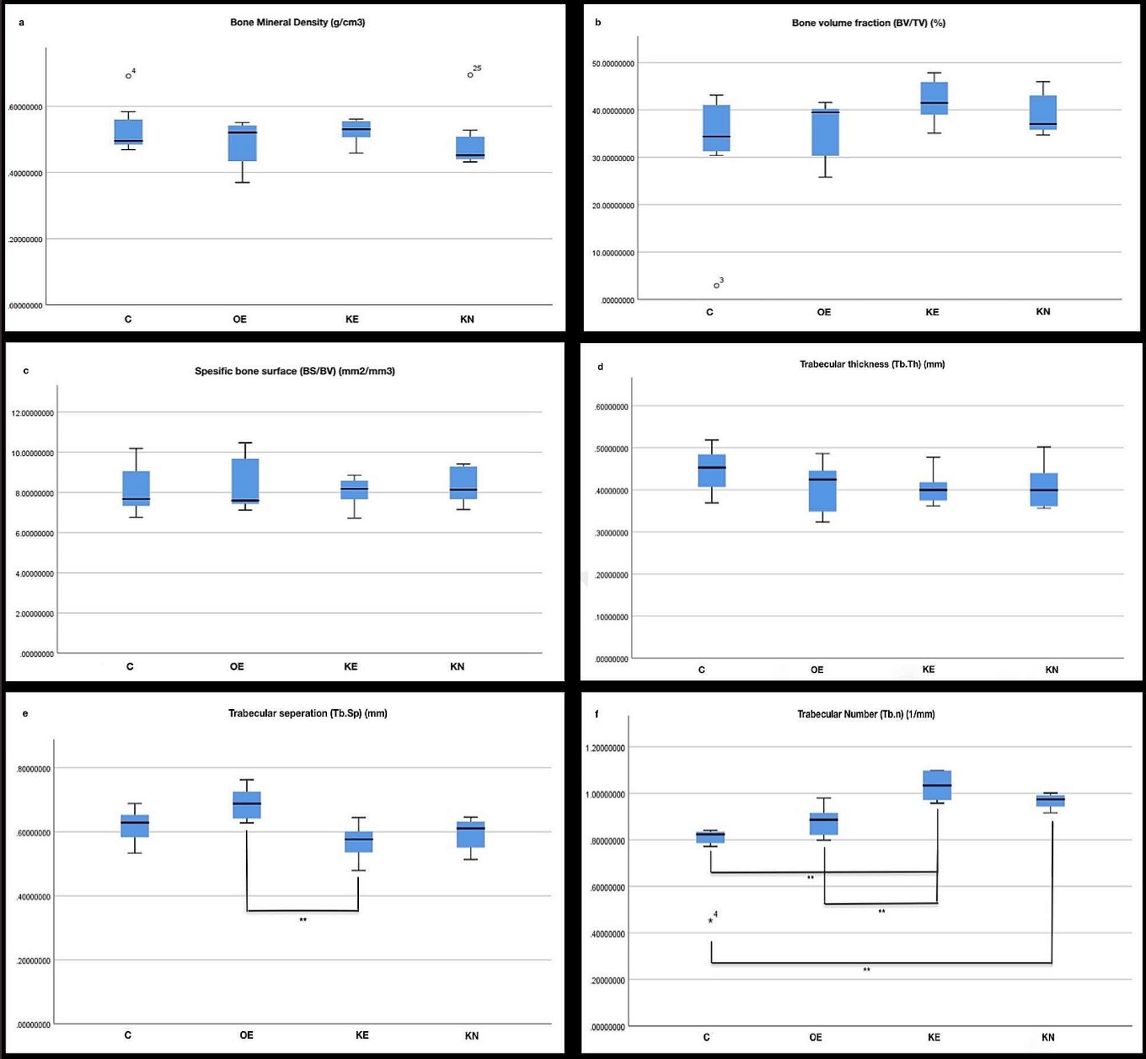
The graphs of bone paramater changes on the maxillary sutural area determined by micro-CT analysis. ** shows statistically significance (Dunn test with Bonferonni *p* < 0.05)

Expansion of mid-palatal suture was observed (Fig. 2). KO supplementation did not cause a statistically significant change in BMD, BV/TV, BS/BV, Tb.Th (Fig. 3a, b, c, d) Paired comparison revealed a statistically significant decrease in Tb.Sp in KE compared with OE. The KN group’s Tb.Sp value was almost statistically significantly lower than OE. While the expansion prosedure increased Tb.Sp, KO supplemantation decreased this effect (Fig. 3e) Paired comparison revealed that the Tb.N of the groups administrated with KO was higher than the C group. KE group Tb.N was found to be statistically significantly higher than the OE group. Tb.N value of the KN was numerically higher than the OE (Fig. 3f) (Table 1.)

**Table 1:** Comparison of micro-CT data between groups

	Bone mineral density (BMD) (g/cm^3^) Mean ± Sd	Bone volume fraction (BV/TV) (%) Mean ± Sd	Spesific Bone surface (BS/BV) (mm^2^/mm^3^) Mean ± Sd	Trabecular thickness (Tb.Th) (mm) Mean ± Sd	Trabecular seperation (Tb.Sp) (mm) Mean ± SS	Trabecular Number (Tb.*n*) (1/mm) Mean ± Sd
C (1)	0.535 ± 0.80	32.137 ± 13.838	8.197 ± 1.312	0.446 ± 0.057	0.617 ± 0.056	0.765 ± 0.140
OE (2)	0.485 ± 0.074	35.419 ± 6.443	8.485 ± 1.399	0.403 ± 0.062	0.687 ± 0.053	0.876 ± 0.066
KE (3)	0.524 ± 0.038	41.781 ± 4.712	8.029 ± 0.768	0.405 ± 0.041	0.569 ± 0.058	1.031 ± 0.063
KN (4)	0.497 ± 0.094	39.337 ± 4.534	8.371 ± 0.972	0.408 ± 0.055	0.590 ± 0.052	0.966 ± 0.34
p*	0.382	0.202	0.969	0.360	0.012*	0.000*
p**					**2–3**	**1–3**,** 2–3**,** 1–4**

*Kruskall-Wallis test *p* < 0.50

**Dunn test with Bonferonni correction *p* < 0.05. The numbers shows the pairwise comparison results between groups

### Histological and immunohistochemical results

#### Interdental distance

**Fig. 4:**
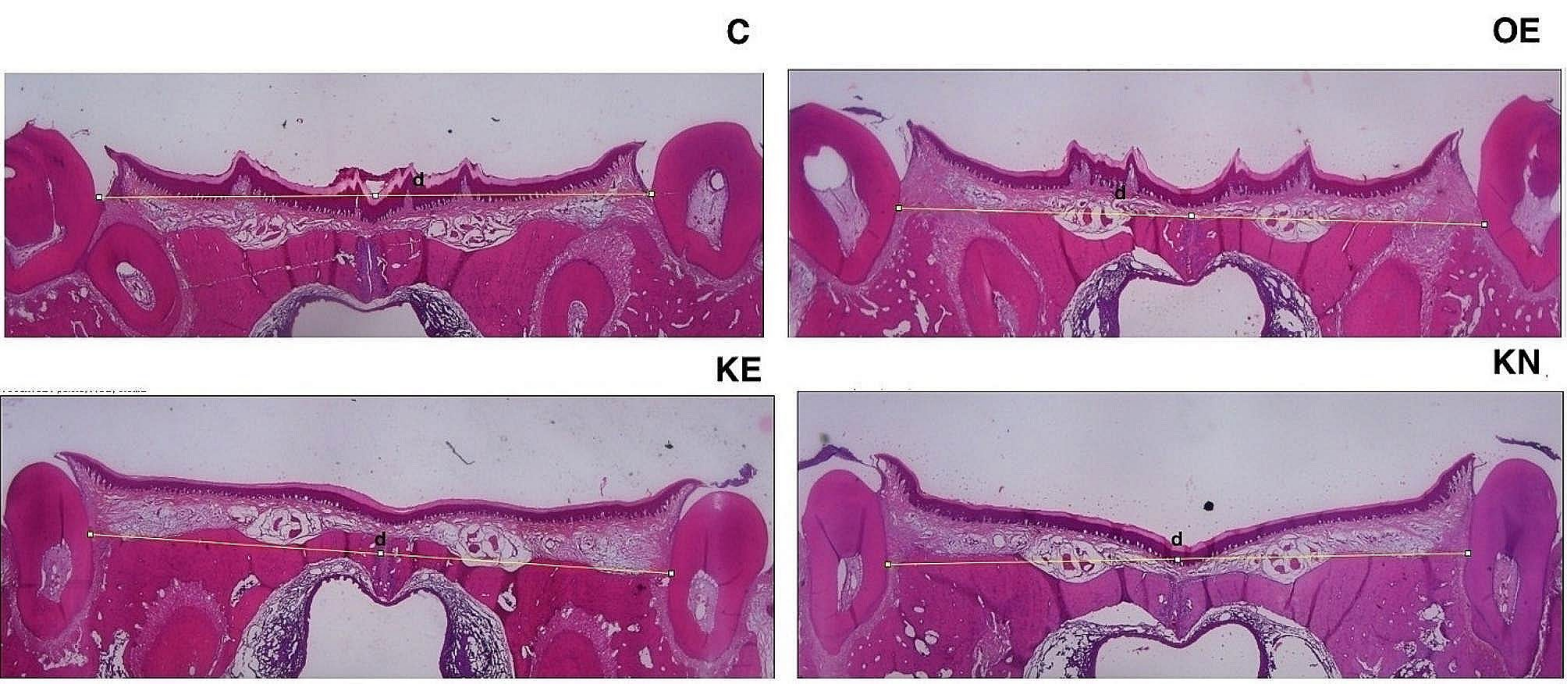
Photomicrographs of histologic sections of sutural areas stained with H&E. d: Measurement of intermolar distance

With expansive force, the mid-palatal suture was expanded, but there was no statistically significant difference between the OE and KE or KN groups. The midpalatal suture width was found the least in KO-administrated groups numerically (Table 2) (Fig. 4)

**Table 2:** Intermolar distance comparison between groups

Molar distance (µm) (d)	Mean ± Sd
C (1)	973.773 ± 23.741
OE (2)	1.049.315 ± 41.079
KE (3)	1.007.897 ± 24.456
KN (4)	1.035.980 ± 30.361
p*	**0.006**
p**	**1–2**

*Kruskall-Wallis test. *p* < 0.050

**Dunn test with Bonferonni correction *p* < 0.05

## Immunohistochemical results

The comprehensive statistical analysis of immunohistochemical staining was shown in Table 3.

**Table 3:** Comparison of immunohistochemical results between groups

	VEGF Mean ± Sd	RANK Mean ± Sd	RANKL Mean ± Sd	OPG Mean ± Sd	TRAP Mean ± Sd
C(1)	0.279 ± 0.187	0.098 ± 0.072	0.339 ± 0.067	0.186 ± 0.149	61.33
OE(2)	0.568 ± 0.061	0.152 ± 0.033	0.322 ± 0.101	0.463 ± 0.212	94.80
KE(3)	0.331 ± 0.207	0.118 ± 0.022	0.175 ± 0.060	1.223 ± 0.790	77.33
KN(4)	0.663 ± 0.326	0.146 ± 0.127	0.263 ± 0.018	1.323 ± 0.241	107.14
p*	0.051	0.098	0.002*	0.000*	0.043*
p**			**2–3**,** 1–3**	**1–3**,** 1–4**	**-**

*Kruskall-Wallis test *p* < 0.050

**Dunn test with Bonferonni correction *p* < 0.05. The numbers shows the pairwise comparison results between groups

**Fig. 5:**
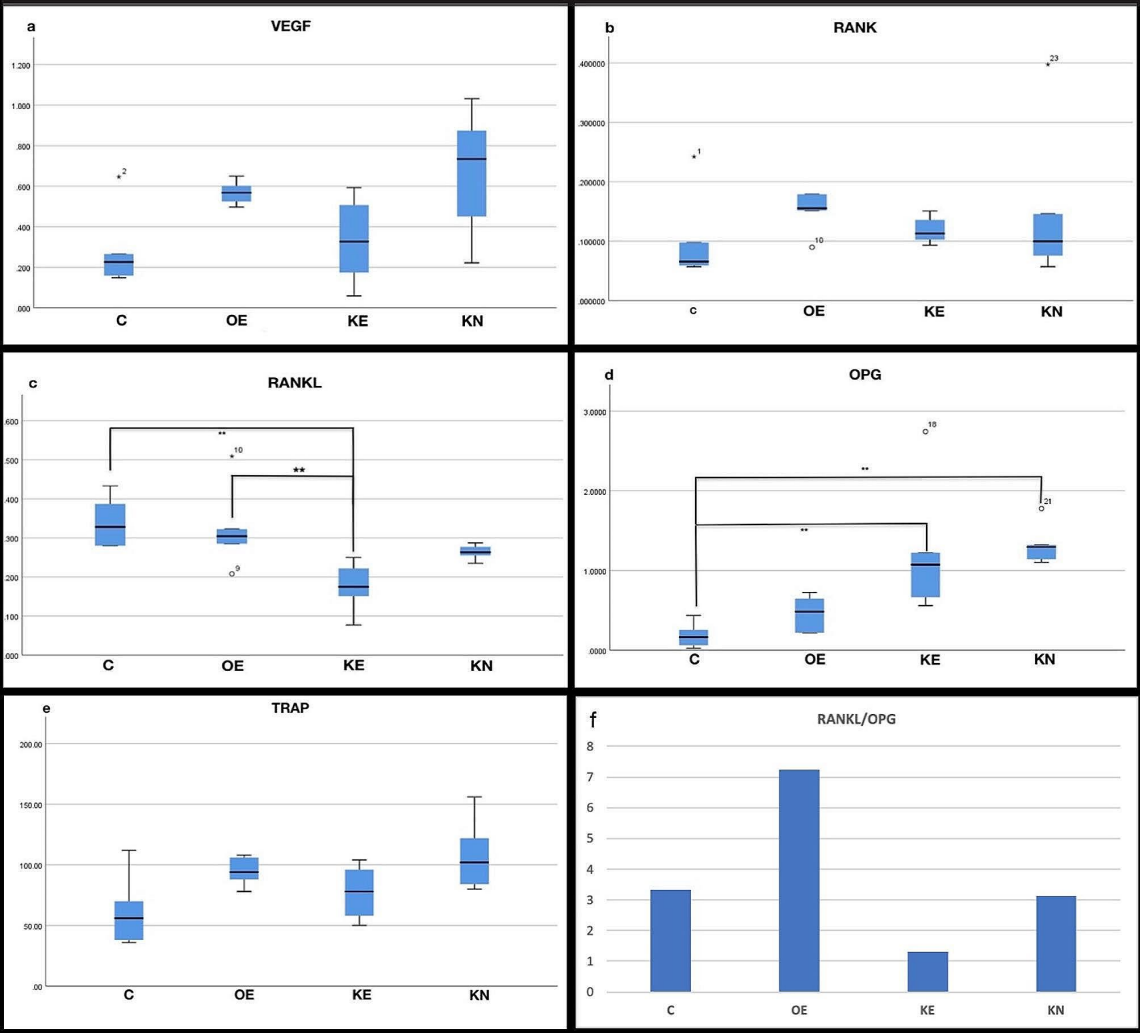
The graphs of immunohistochemical changes on the maxillary sutural area. ** shows statistically significance (Dunn test with Bonferonni *p* < 0.05)

**Fig. 6:**
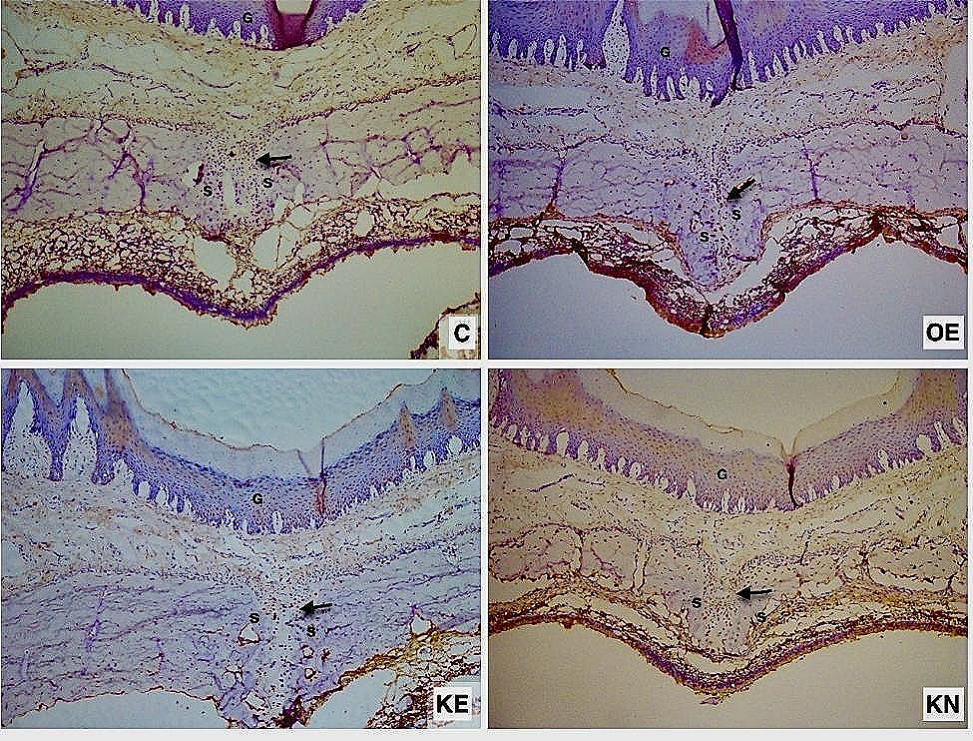
Photomicrographs of histologic sections of sutural areas stained with VEGF antibody. Positive cells in the osteogenic zone (arrow). S: Sutural connective tissue. G: Gingiva

There was almost a significant difference between the groups in terms of VEGF. The RME procedure increased VEGF. During the experimental period, KO supplementation decreased the amount of VEGF compared to the OE group. However, KO supplementation during the nursery and experimental period increased the amount of VEGF (Fig. 5a) (Fig. 6).

**Fig. 7:**
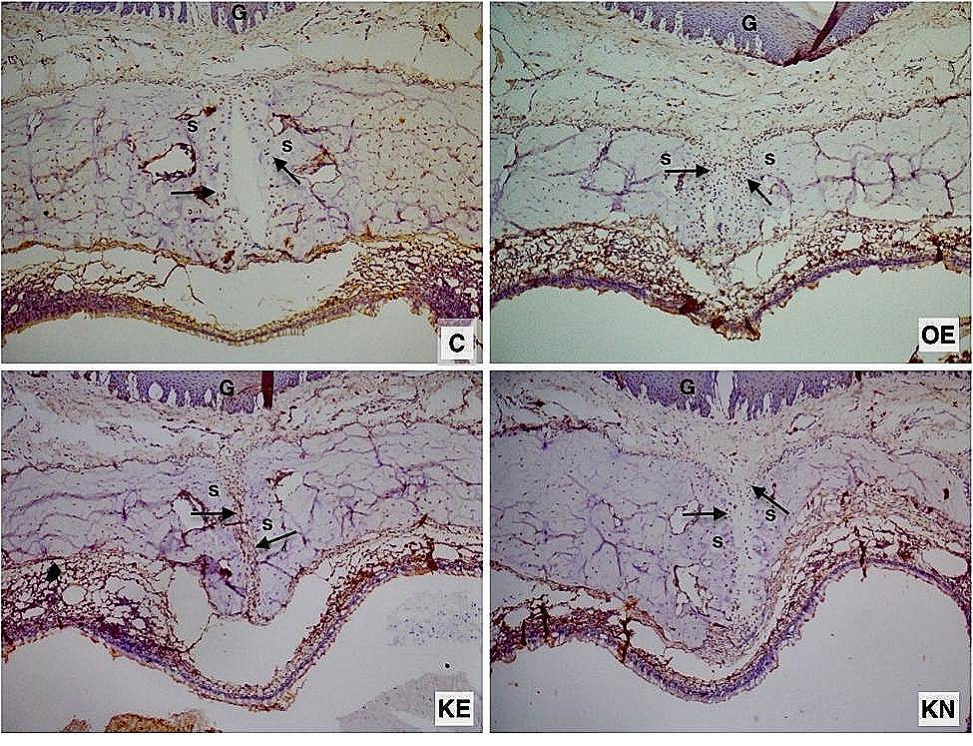
Photomicrographs of histologic sections of sutural areas stained with RANK antibody. Positive cells in the osteogenic zone (arrow). S: Sutural connective tissue. G: Gingiva

There was no significant difference between the groups in terms of RANK, showing that antibody staining intensity of the expansion groups was numerically higher than the C. KO supplemented group’s RANK value was numerically lower than OE (Fig. 5b) (Fig. 7)

**Fig. 8:**
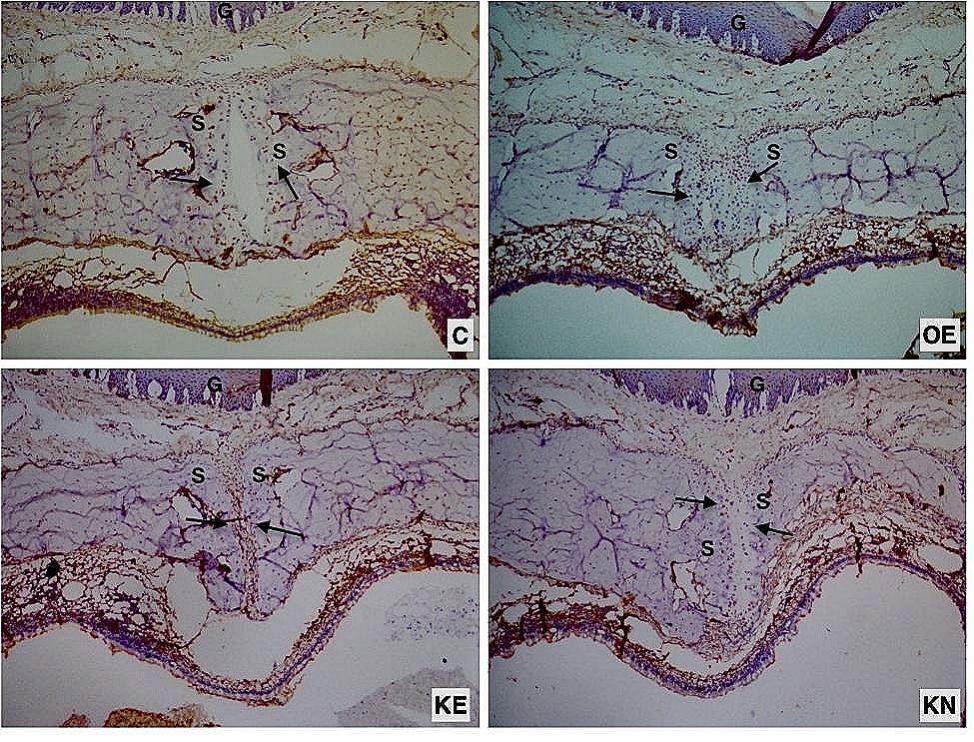
Photomicrographs of histologic sections of sutural areas stained with RANKL antibody. Positive cells in the osteogenic zone (arrow). S: Sutural connective tissue. G: Gingiva

RANKL results showed a significant difference between groups. During the experimental period, KO decreased the RANKL level compared to the OE group. Supplemantation with KO during the nursery period kept the RANKL level numerically higher than the KE group (Fig. 5c) (Fig. 8.)

**Fig. 9:**
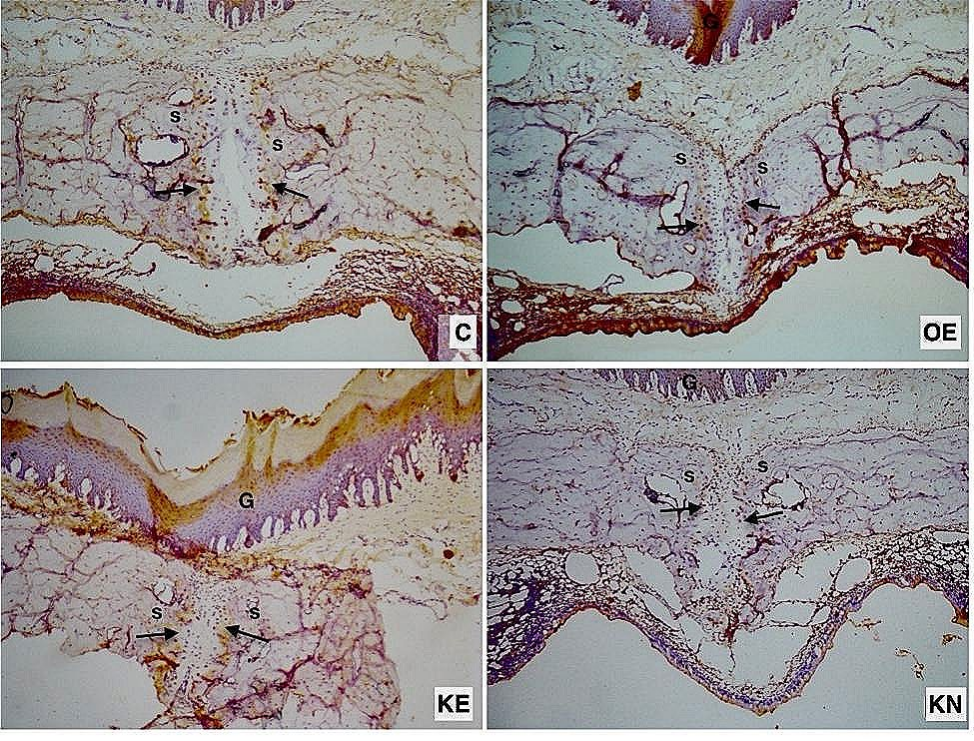
Photomicrographs of histologic sections of sutural areas stained with OPG antibody. Positive cells in the osteogenic zone (arrow). S: Sutural connective tissue. G: Gingiva

There was a significant difference between the groups in terms of OPG. In the pairwise comparison, statistically significantly higher OPG staining was observed in the KO supplemented groups compared to the C, and numerically higher than OE. During the nursery period, KO supplementation further increased the amount of OPG (Fig. 5d) (Fig. 9)

**Fig. 10:**
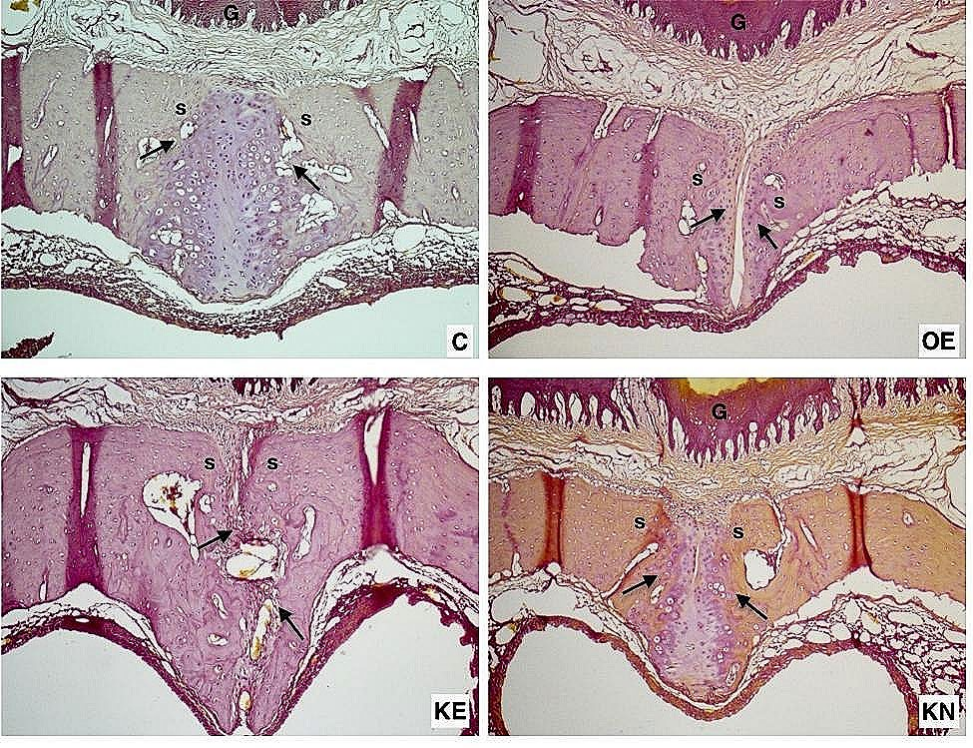
Photomicrographs of histologic sections of sutural areas stained with TRAP antibody. Positive cells in the osteogenic zone (arrow). S: Sutural connective tissue. G: Gingiva

There was a significant difference between the groups in terms of TRAP data. The RME procedure increased TRAP. During the expansion and retention period, KO supplementation decreased the amount of TRAP compared to the OE. KO supplementation during the nursery period increased the amount of RANKL and the most TRAP was found in KN group. However, pairwise comparison analysis did not show any significant difference (Fig. 5e) (Fig. 10).

Although RANKL/OPG results showed no significant difference between groups, KE and OE showed the lowest and highest value, respectively (Fig. 5f) (Table 4)

**Table 4:** Comparisons of the groups in terms of RANKL/OPG ratio

	C	OE	KE	KN	p*
	Mean *±* SD	Mean *±* SD	Mean *±* SD	Mean *±* SD	
RANKL/OPG	3.32 ± 4.21	7.23 ± 10.43	1.32 ± 1.18	3.13 ± 4.81	0.661

## Discussion

The surgical or orthopedic procedures to improve maxillary bone volume and contour are primarily important for the patients seeking an esthetic and healthier orofacial function [17, 18]. In maxillary expansion cases, to preserve gained bone volume, many researchers have tried various methods to enhance regeneration potential in the mid-palatal suture. This would increase the popularity of expansion treatment in orthodontic patients and introduce new perspectives on integrating surgical and orthodontic treatments. In this context, Antarctic KO has been receiving increasing attention due to its nutritional and functional potentials. Besides their anti-inflammatory and anti-resorbtive effects, n-3 PUFAs are also known to have anti-oxidant properties because of astaxanthin constitution that might deal with the reactive oxygen species. Astaxanthin is 54 times more potent as an antioxidant than β-carotene, and 100 times more potent than α-tocopherol [13, 19].

Advances in micro-CT images, which provide high resolution and accuracy, have encouraged the utilization of these method for the estimatiing RME and quantifying radiographic changes via the differences in pixel values. BMD is a biomarker that measures the amount of mineral per unit bone volume and significantly affects bone strength. Bone volume decreasing in the mid-palatal area after RME to the minimum and increasing with bone formation over time has been reported [20]. In the present study, BMD values revealed similar results with the study [20], reporting the pattern of BMD decreasing with RME in expansion groups. Even if statistically non-significant, KO administration in both the experimental and nursery periods recovered the loss of BMD. Additionally, the result of the immunohistochemical examination not being supported by BMD and BV/TV findings can be explained by the micro-CT detection algorithm [21]. The regions of interest for the micro-CT were equidimensional cuboids starting from the midpalatal bony edges. Because a uniform threshold value was applied to identify bone tissue, newly formed bone at the edges of the midpalatal bone, which is relatively low in mineralization, might not have been recognized as bone tissue. Consequently, the lack of significant differences between groups in terms of BMD and BV/TV could be due to the presence of low-mineralized bone in the analyzed regions.

The Tb.Sp and Tb.N analysis showed statistically significant quantity difference between OE-KE groups, emphasizing that KO stimulate new bone formation at the end of retention period. Based on the statistical results, it is observable that KO supplement yielded the highest Tb.N and lowest Tb.Sp, suggesting a greater amount of bone accumulation in the KN and KE compared to the OE.

The results of the present study indicated that the KO did not affect the displacement of the maxillary halves of rats that underwent RME. However, the midpalatal suture expansion was numerically less in KO-administrated groups. The following explanation contextualises this finding: It is well known that suture remodeling is linked to local strains. Within the local mechanical environment, osteogenesis was promoted, leading to new bone formation at the suture edges. This may have caused the suture width to decrease in the KO and KN groups after RME. The narrowing of the suture in the KO following expansion may indicate that more bone had formed and filled the suture gap, thereby reducing its width.

RME involves a kind of tissue damage, and hypoxia is observed in mid-palatal sutures in the early stages of expansion. After RME, several reactions begin similar to those observed in wound healing processes, involving angiogenic growth factors, with VEGF being the most important angiogenesis mediator regulated by hypoxia, released and upregulated in the palatal suture tissue [22]. Studies also indicated that VEGF, produced from osteoblast precursors and highly expressed by mechanical stimuli, enhances the number of osteoclasts and osteoblast as a paracrin factor [23]. During RME, VEGF levels increased significantly, decreases over time, but do not return the baseline values [22]. Zhuang et al. [24] reported that DHA and EPA suppreses VEGF mRNA expression and have anti-angiogenic effect. This view is also supported by Ogrenim et al. [10], who reported that proinflamatuary markers such as RANKL, IL-6, IL-1β andVEGF, and accordingly the amount of tooth movement, were significantly lower in omega-3-administrated rats. In the present study, although VEGF staining did not show significant difference between groups, the p value was close to the level of significance when comparing the C and expansion groups across the evaluation periods. KO decreased the amount of upregulateted VEGF compared with OE group. However, prolonged KO administration during the nursery period significantly increased the amount of VEGF.

In the present study, since RME is a process of bone remodeling managed by osteoblasts and osteoclasts, the distribution and quantity of these cells were analyzed by RANK/RANKL/OPG and TRAP staining to determine the state of bone remodeling in different groups. Early osteogenesis markers (RANK and OPG) were highly expressed in the expanded palatal sutures, while they were relatively low in C group. The non-significant lowest expression of RANK and RANKL was considered as an indicator of completion of early osteogenesis in KE group. The ratio of red-stained osteoclasts was primarily found at the edge of bone trabeculae and to a lesser extent in the non-osseous fibrous tissue. Semi-quantitive TRAP analysis indicated fewer osteoclasts in the C and KE groups, suggesting that KO administration completed the process of bone remodelling after 17 days. The KN group showed the highest number of osteoclasts, verifying more active remodeling, while the number of osteoclasts in OE was lower when compared to KN. At first glance, this could indicate that prolonged KO usage might postpone the bone remodelling process. Also, the reduced VEGF score in KE is an important predictors of using KO during the experimental period to enhance bone remodelling. Contrary to the KE group, the continued increase of VEGF in the KN group is a marker of ongoing angiogenesis. Non-significant upregulation of VEGF in KN may be interpreted as the usa of KO in the nursery period could give rise to a postponement of bone remodelling. However, neovascularization is another key mechanism involved in wound healing, and it was reported that KO activates the cutaneous expression of VEGF, confirming that KO enhanced neoangiogenesis in wounds [25]. This non-significant increase in VEGF may also be an indicator of neovascularization that will attract growth factors, osteoblasts and osteoclasts to scar tissue resulting from RME. In the C group, VEGF was expressed with no signs of inflammation, suggesting that it was also produced in healthy dental tissue. This VEGF protein expression would be indicative of physiological angiogenesis and plays an important role in maintaining homeostasis, corroborating the findings of VEGF in the present research [26]. In parallel, VEGF and osteoclastogenesis indicators (RANK and TRAP) appeared in correlation, showing that KO usage during the experimental period supports bone formation in the midpalatal suture. These immunohistochemical results indicated expansive stress-initiated distraction osteogenesis based on intramembranose osteogenesis process in the mid-palatal suture.

In previous studies, the RANKL/OPG was employed to indicate the equilibrium between osteoclastic activity and osteogenesis. A greater RANKL/OPG ratio indicated a prevalence of osteoclast formation, whereas a lower ratio indicated osteogenesis [27]. Among the expansion groups, OE showed the highest expression of RANKL, which was similar to the trend of RANK staining, while OPG expression was lowest in OE. By comparing the relative value of RANKL/OPG, an obvious trend could be observed that the value in KE was lower than in the OE and C groups, indicating that KO promoted bone formation within the mid-palatal suture and benefited the balance of bone remodeling towards osteogenesis. The very close value of KN to C is interpreted as completed process of bone formation.

While this study provides valuable insights into the effects of KO supplementation on bone formation in the midpalatal suture following RME, some limitations should be acknowledged. First, the sample size of 28 rats, although sufficient for preliminary observations, is relatively small and may limit the generalizability of the findings. Second, the duration of the study, comprising a 5-day expansion followed by a 12-day retention period, provides a limited timeframe for observing the long-term effects of KO supplementation on bone remodeling and stability. Future studies with extended observation periods are necessary to fully understand the potential benefits and limitations of KO in the context of RME. Furthermore, while the use of micro-CT, histological staining, and immunohistochemical analysis provides a comprehensive assessment of bone formation and architecture, additional biochemical and molecular analyses could offer deeper insights into the underlying mechanisms through which KO influences bone metabolism. Additionally, mechanical testing of the regenerated or newly formed bone using a device such as a universal testing machine to compare the mechanical properties of new bone among different groups.

## Conclusion

In this adopted rat RME model, the combination of findings provides significant support for the conceptual premise that the administation of KO during the expansion period or starting from before RME procedure could potentially reduce relapse and improve osteogenesis within mid-palatal suture. The most important evidence supporting this claim is reduced RANKL/OPG ratio, increased Tb.N and reduced Tb.Sp values. The only exception is the VEGF values, which are confusing and open to debate regarding the KO administration interval. This work provides a comprehensive snapshot of KO activities on RME by being the first study in the literature so far. A relatively large sample and the extended duration of the study will strongly enhance the generalizability of the findings and allow ample time for observing the long-term effects of KO supplementation on bone remodeling and stability.

## Data Availability

The datasets used and/or analyzed during the current study are available from the corresponding author on reasonable request.

## Abbreviations

KO: Krill Oil
C: Control
KE: Krill Expansion (supplement during the expansion and retention)
OE: Only Expansion (no supplement but expansion and retention)
KN: Krill Oil Nursery (supplement during nursery phase of 40 days and during the expansion and retention)
RME: Rapid Maxillary Expansion
Micro-CT: Micro-Computed Tomography
BMD: Bone Mineral Density
BV/TV: Bone Volume/Total Volume
BS/BV: Bone Surface/ Bone Volume
Tb.N: Trabecular Number
Tb.Th: Trabecular Thickness
VEGF: Vascular Endothelial Growth Factor
H-SCORE: Histochemical Scoring
RANKL/OPG: Receptor Activator Nuclear Kappa B Ligand/Osteoprotegerin
PUFAs: Poly Unsaturated n-3 Fatty Acids
EPA: Eicosapentaenoic Acid
DHA: Docosahexaenoic Acid
DAB: 3,3’-diaminobenzidine

## References

[1] Oppenheim A. The crisis in orthodontia part I. tissue changes during retention. Int J Orthod. 1934;20:639–44.

[2] Vali S, Khosravani S, Nobar BR, Motamedian SR. Rapid maxillary expansion supplementary methods: a scoping review of animal studies. Int Orthod. 2022. 10.1016/j.ortho.2022.100614. 35153159 10.1016/j.ortho.2022.100614

[3] Cannavale R, Chiodini P, Perillo L, Piancino MG. Rapid palatal expansion (RPE): Meta-analysis of long-term effects. Orthod Craniofac Res. 2018;21:225–35. 30207637 10.1111/ocr.12244

[4] Zimring JF, Isaacson RJ. Forces produced by rapid maxillary expansion 3. Forces present during retention. Angle Orthod. 1965;35:178–86. 14331018 10.1043/0003-3219(1965)035<0178:FPBRME>2.0.CO;2

[5] Gok GD, Topbasi NM, Baydas B, Uslu H, Ceylan I, Yavuz I, et al. Effects of rapid maxillary expansion on the temporomandibular joint: a bone scintigraphy study. Turk J Orthod. 2021;34:176–81. 35110188 10.5152/TurkJOrthod.2021.20162 PMC8939251

[6] Garcia Costa J, Galindo TM, Mattos CT, De A, Cury-Saramago A. Retention period after treatment of posterior crossbite with maxillary expansion: a systematic review. Dent Press J Orthod. 2017;22:35–44. 10.1590/2177-6709.22.2.035-044.oar PMC5484268 28658354

[7] Irgin C, Corekci B, Ozan F, Halicioglu K, Toptas O, Birinci Yildirim A, et al. Does stinging nettle (Urtica dioica) have an effect on bone formation in the expanded inter-premaxillary suture? Arch Oral Biol. 2016;69:13–8. 27209059 10.1016/j.archoralbio.2016.05.003

[8] Halicioʇlu K, Çörekçi B, Akkaş I, Irgin C, Özan F, Yilmaz F, et al. Effect of St John’s wort on bone formation in the orthopaedically expanded premaxillary suture in rats: a histological study. Eur J Orthod. 2015;37:164–9. 24997024 10.1093/ejo/cju028

[9] Gad AM, Soliman SO. Evaluation of systemic Omega-3 PUFAs effect on orthodontic tooth movement in a rabbit model: RCT. Angle Orthod. 2023;93:476–81. 36928563 10.2319/110222-750.1 PMC10294573

[10] Ogrenim G, Cesur MG, Onal T, Kara M, Sirin FB, Yalcin GD, et al. Influence of omega-3 fatty acid on orthodontic tooth movement in rats: a biochemical, histological, immunohistochemical and gene expression study. Orthod Craniofac Res. 2019;22:24–31. 30447132 10.1111/ocr.12253

[11] Karunia D, Sripudyani P, Mubarika S, Widyarini S. Effects of docosahexaenoic acid (dha) microalgae (r) on orthodontic tooth movement in the New Zealand white rabbit. J Int Dent Med Res. 2019;12:1287–92.

[12] Xie D, Gong M, Wei W, Jin J, Wang X, Wang X, et al. Antarctic krill (Euphausia superba) oil: a comprehensive review of chemical composition, extraction technologies, health benefits, and current applications. Compr Rev Food Sci Food Saf. 2019;18:514–34. 33336946 10.1111/1541-4337.12427

[13] Kim MG, Yang I, Lee HS, Lee JY, Kim K. Lipid-modifying effects of krill oil vs fish oil: a network meta-analysis. Nutr Rev. 2020;78:699–708. 32073633 10.1093/nutrit/nuz102

[14] Colletti A, Cravotto G, Citi V, Martelli A, Testai L, Cicero AFG. Advances in technologies for highly active omega-3 fatty acids from krill oil: clinical applications. Mar Drugs. 2021;19. 10.3390/md19060306 PMC8226823 34073184

[15] Nair A, Jacob S. A simple practice guide for dose conversion between animals and human. J Basic Clin Pharm. 2016;7:27. 27057123 10.4103/0976-0105.177703 PMC4804402

[16] USFDA. Guidance for Industry: Estimating the maximum safe starting dose in initial clinical trials for therapeutics in adult healthy volunteer. US Food and Drug Administration. 2005. https://www.fda.gov/media/72309/download. Accessed 19 May 2024.

[17] Aldelaimi TN, Khalil AA. Reconstruction of facial defect using deltopectoral flap. J Craniofac Surg. 2015;26:e786–8. 26595007 10.1097/SCS.0000000000002056

[18] Aldelaimi TN, Khalil AA. Maxillary sinus augmentation. J Craniofac Surg. 2016;27:e557–9. 27428922 10.1097/SCS.0000000000002864

[19] Shah MMR, Liang Y, Cheng JJ, Daroch M. Astaxanthin-producing green microalga haematococcus pluvialis: from single cell to high value commercial products. Front Plant Sci. 2016. 10.3389/fpls.2016.00531. 27200009 10.3389/fpls.2016.00531 PMC4848535

[20] Takenouchi H, Mayahara K, Arai Y, Karasawa Y, Shimizu N. Longitudinal quantitative evaluation of the mid-palatal suture after rapid expansion using in vivo micro-CT. Arch Oral Biol. 2014;59:414–23. 24534134 10.1016/j.archoralbio.2014.01.010

[21] Cheng Y, Sun J, Zhou Z, Pan J, Zou S, Chen J. Effects of lactoferrin on bone resorption of midpalatal suture during rapid expansion in rats. Am J Orthod Dentofac Orthop. 2018;154:115–27. 10.1016/j.ajodo.2017.09.020 29957309

[22] Stuani AS, Silvano PRÁ, Arnez MFM, Mira PC da S, Gorita MC, Monteiro PM, et al. VEGF and FGF-2 released in palatal suture after rapid maxillary expansion (RME). Braz Dent J. 2021;32:98–103. 33914010 10.1590/0103-6440202103527

[23] Kohno S, Kaku M, Tsutsui K, Motokawa M, Ohtani J, Tenjo K, et al. Expression of vascular endothelial growth factor and the effects on bone remodeling during experimental tooth movement. J Dent Res. 2003;82:177–82. 12598545 10.1177/154405910308200306

[24] Zhuang W, Wang G, Li L, Lin G, Deng Z. Omega-3 polyunsaturated fatty acids reduce vascular endothelial growth factor production and suppress endothelial wound repair. J Cardiovasc Transl Res. 2013;6:287–93. 22993129 10.1007/s12265-012-9409-0

[25] Hao W, Meng H, Li H, Zheng Y, Song C, Jiang Z, et al. Local application of krill oil accelerates the healing of artificially created wounds in diabetic mice. Nutrients (MDPI). 2022;14:4139. 10.3390/nu14194139 PMC9571309 36235791

[26] Prapulla DV, Sujatha PB, Pradeep AR. Gingival crevicular fluid VEGF levels in periodontal health and disease. J Periodontol. 2007;78:1783–7. 17760549 10.1902/jop.2007.070009

[27] Arnez MFM, Ribeiro LSN, Barretto GD, Monteiro PM, Ervolino E, Stuani MBS. RANK/RANKL/OPG expression in rapid maxillary expansion. Braz Dent J. 2017;28:296–300. 29297549 10.1590/0103-6440201601116

